# Searching for meaning in a post-scarcity society. Implications for creativity and job design

**DOI:** 10.3389/fpsyg.2023.1198424

**Published:** 2023-09-12

**Authors:** Vittorio Pelligra, Pier Luigi Sacco

**Affiliations:** ^1^Department of Economics and Business, University of Cagliari, Cagliari, Italy; ^2^Department of Philosophical, Pedagogical and Economic-Quantitative Sciences, University “G. D’Annunzio” of Chieti-Pescara, Pescara, Italy; ^3^ISPC-CNR, Naples, Italy

**Keywords:** meaning, post-scarcity, well-being, productivity, creativity, job design

## Abstract

The significance of meaningful and productive work, and the search for profound meaning within it, is akin to the air we breathe. Its importance is often realized only when it becomes contaminated or depleted. In contemporary societies, there is a growing awareness of the significance of the meaning of work, while simultaneously witnessing mounting mistrust and disillusionment as to the significance and social value of numerous jobs. There is paradoxically an increasing demand for meaningful work, while the supply of such work appears to be gradually decreasing. At present, we are recognizing the importance of this vital component that sustains our well-being as it begins to dwindle. The absence of meaningful work may stem from the nature of the work itself, the organizational environment in which it takes place, the prevailing corporate culture, or even the way in which tasks are defined and managed, which makes it challenging to find a sense of purpose and meaning in what we do. While progress can be made on both fronts, addressing cultural and organizational aspects is a more expedient means of intervention without the need of waiting for structural changes in the global economic and social systems.

## 1. Meaning and post-scarcity society

A post-scarcity society refers to a social order where the prospect of basic threats to survival, such as starvation, due to a scarcity of resources is generally not considered likely enough to shape daily choices. The actual transition to a post-scarcity society began in the post-World War II period, starting from North America (Lhamon, 2002) and gradually extending to Europe, Australia, and other non-Western industrialized countries like Japan and, subsequently, South Korea (Sadler, 2010).

In several countries, the transition to a post-scarcity society has not fully occurred yet, and different regions may currently sit at opposite sides of the transition spectrum. For example, in China, the most developed metropolitan areas and provinces reside fully in the post-scarcity sphere, whereas rural and remote provinces are still heavily influenced by scarcity and consider extreme poverty and starvation as a tangible possibility, with consequential differences in prevailing notions and goals of life satisfaction (Wu, 2022).

The transition to a post-scarcity society has far-reaching implications for human attitudes towards both tangible and intangible resources and, more generally, the concept of resourcefulness. In a scarcity society, accessing resources is problematic and fundamental to survival, and achieving such access is purposeful (Skidelsky and Skidelsky, 2013). The ability to obtain the resources required for survival is an indication of a meaningful life and having achieved a purpose. However, in post-scarcity societies, obtaining resources for survival is no longer a meaningful purpose in itself, as it is now perceived as relatively unproblematic. Life’s worth is determined by what happens after securing the resources needed for survival.

Although in scarcity societies, the pursuit of meaning is not solely dependent on acquiring resources for survival, the experience of meaning as a transgression of the laws of scarcity is typically confined to limited occasions, such as festive times, and is in turn conducive to better social capacities to overcome scarcity (Hayden, 2009). During festive times, scarcity societies create sophisticated rituals that focus on the shared pursuit of meaning, where even survival threats and needs are symbolically encapsulated in a wider, encompassing imaginary. Although resourcefulness is vital to making life worth living, these rituals suggest that there is a further sphere of meaning in which survival itself reflects underlying motives and becomes part of an orderly, harmonious state of things that transcends individual experience, provides real societal cement, and defines collective identity (Xygalatas, 2022).

The primary difference with post-scarcity societies is that the distinction between time devoted to survival needs and time left for the pursuit of meaning gradually fades away. If survival is no longer problematic, humans expect to find a deeper meaning in activities that were previously legitimized by their mere contribution to satisfying survival needs (Aguiar, 2011). This is particularly evident in the case of work experience. In a scarcity society, having a job is meaningful because it can provide the resources for survival. However, in a post-scarcity society, even a well-paying job that is not perceived as intellectually, emotionally, and socially fulfilling can be regarded as meaningless (Lencioni, 2007). This phenomenon has become increasingly apparent after the pandemic crisis, with the emergence of a new social trend of people quitting “quality” jobs (according to the standard criteria of a scarcity society) to seek a better existential balance between resource acquisition, quality of life, and the pursuit of meaningful goals (Peters et al., 2022).

## 2. Happiness vs. meaningfulness

The concept of quality of life in the context of a post-scarcity society has often been linked to the notion of happiness, which is generally accepted as an appropriate measure of the psychological state in which individuals feel that their life is worthwhile. As a result, happiness indexes are now regularly calculated at national levels to determine the extent to which countries are providing their citizens with satisfactory living conditions that go beyond the mere provision of resources. Such happiness-related measures can be seen as post-scarcity counterparts of traditional welfare measures based on economic value creation such as GDP-based ones.

Needless to say, the topics of happiness and related ones such as well-being, life satisfaction and quality of life, to name just a few, have been the object of endless analysis and speculation throughout human intellectual history, and there is hardly any philosophical system which does not contain or imply a specific conceptualization on such issues. A few useful references for the interested reader are, among many others, the books of Haidt (2006), White (2006), and Feldman (2010). Among the many authors who have specifically problematized the notion of happiness in the context of the workplace, it is worth to briefly mention at least Karl Marx’s theory of alienation, which ultimately sees the possibility of purposefulness in human activity only after the final overcoming of the exploitative relations embedded in the capitalist socio-economic order as enabled by socialism (Byron, 2013), and Max Weber’s notion of disenchantment as a crucial gulf, again characteristic of the capitalist socio-economic order, between the dimension of playful pleasure as a deep motivator of human action and that of labor productivity as an essentially technical, instrumental activity (Sommer and Sacco, 2019). More generally, there is a tendency in foundational philosophical thinking to conceive of work as remote from, if not antithetical to, the sphere of happiness, in line with the classical Biblical identification of work as labor and suffering as a consequence of human sinful nature (Ellul, 1985). Therefore, it has become commonplace to consider happiness and wellbeing as goals to be pursued outside of the work sphere, typically in relation to leisure and “free time” (again, a telling expression), and to consider work as a liability and not an asset in the balance sheet of well-being and quality of life.

If, according to this view, misery comes from oppressive social relations, happiness must therefore be sought in individual, redeemed time. Consequently, happiness is essentially conceived of as an individualistic and hedonic construct in most economically advanced, post-scarcity societies (Joshanloo and Jarden, 2016). In such cultural contexts, to achieve happiness, individuals must act on their internal states and regulate their affect to be positively and not negatively valenced, in a stable and robust manner, but seeking happiness at all times rather than seeking it at the right time may be self-defeating (Tamir and Ford, 2012). And while interacting with others can greatly contribute to an individual’s happiness, a prevalently individualistic and hedonic orientation toward happiness makes interaction instrumental to regulating one’s own affective valence and fulfilling materialistic goals (Ozimek et al., 2017). In post-scarcity societies, the happiness drive often builds upon the individual rather than the collective dimension, and it is not surprising that the pursuit of happiness as hedonistic self-indulgence has been a driving force behind post-industrial consumerism (Acerbi and Sacco, 2022), which is the root cause of much social injustice and environmental degradation (Princen et al., 2002). If pursuing happiness is about avoiding anything that can threaten it, as a result, it is not a socially adaptive goal that can help us harness our cooperative problem-solving skills and therefore our capacity to collectively respond to social challenges in highly organized, effective ways. To harness such skills and capacities, we need to think and act in terms of collectively satisfactory lifetime goals, rather than simply seeking individual happiness. In this regard, it is important to introduce another notion that, like happiness, is commonly associated to quality of life and wellbeing, but with very different implications: flourishing. Providing a critical comparative discussion of the differences between happiness and flourishing is well beyond the scope of the present paper, also in view of the long traditions of thought behind each notion; see Compton and Hoffman (2019) for a comprehensive analysis. However, some useful distinctions can be introduced here. Flourishing involves developing human potential fully by taking advantage of all possible enabling conditions, including individual, social, and environmental factors (VanderWeele, 2017). Unlike happiness, flourishing is not a state but a process, and it cannot be achieved alone (Johnson, 2022). Humans cannot flourish alone because social exchange is a basic premise of human development (Gauvain and Perez, 2015). The flourishing of one individual is also an enabling condition for the flourishing of others, unlike happiness, which can make others feel more miserable in comparison. Seeing someone else happy may be felt as a relative deprivation (they have something I do not have) but seeing someone flourish is an inspiration (they are doing something I could do myself). Flourishing is therefore a socially self-catalyzing process (Nelson et al., 2016) that can empower our societies to harness successful large-scale cooperation by honing individual undisclosed potential through mutual fertilization. Moreover, flourishing may also lead to happiness as a side outcome.

In a nutshell, one could argue that flourishing is an essentially less individualistic and self-centered notion of wellbeing than happiness, and that as a consequence it has deeper and more long-lasting implications for human sociability. Flourishing together turns out to be more fulfilling than being happy alone, and this in turn implies focusing more on the happiness of others than on one’s own (Titova and Sheldon, 2022). Moreover, unlike happiness, flourishing is deeply related to meaningfulness (Yeoman, 2021). Happiness is often depicted in popular media as the absence of any concern and focus on instantaneous pleasurable conditions. However, thinking of happiness as the ultimate goal of human existence in such terms is concerning, as a life completely devoid of challenges and goals is hardly attractive for human psychology. Experiencing failure and having to hone one’s own personal resources to obtain a highly sought-after outcome is exciting and rewarding (Wiese, 2007), and this is the reason why humans are so interested in apparently senseless activities such as competitive sports (Thedin Jakobsson, 2014). Meaningfulness is a much better descriptor of the conditions under which humans feel their life is worth living, and flourishing is the natural companion to meaningfulness in terms of how the pursuit of meaning is made possible by the development of one’s human potential.

## 3. Determinants of meaning

“Man is equally incapable of seeing the nothingness from which he emerges and the infinity in which he is engulfed.” writes Blaise Pascal in his *Pensées* (1670). Perhaps it is to escape this horrifying polarity that it is so necessary for us to find meaning in our lives; to exorcise the dimensions of nothingness and infinity, so concrete and, at the same time, incomprehensible. This is also why it may be useful to start thinking about the prospect of an “economy of meaning,” which places at the center of human action, even in the economic sphere, that complex process of seeking and constructing a profound meaning of existence, and to do so starting precisely from the role that work plays in this quest, and how organizations can facilitate or hinder the process of meaning recognition (Rosso et al., 2010). We then begin to ask ourselves, going a little more to the heart of the question, what are the tools and strategies that we can use to grasp, at least a little, the meaning of our existence.

“Making sense,” in one of the main meanings of the expression, essentially stands for ‘telling a story’ discovering the plot of our life, its main characters and supporting actors, the antecedents, the turning points, the twists, the growing tension that is hopefully directed towards a decisive happy ending. As Gilovich (1991) reminds us: “We are predisposed to see order, pattern, and meaning in the world, and we find randomness, chaos, and meaninglessness unsatisfying. Human nature abhors a lack of predictability and the absence of meaning.” (p. 13). That’s why we tell each other stories: to give meaning to the complexity of experience because that’s how our brain works (Berns, 2022). When asked to describe ourselves or a friend, it is likely that one would begin by sharing a narrative or anecdote about the person in question, including how we met and the experiences that have shaped our identities. The question “who am I?” can only be answered in the form of a story that integrates the defining aspects of our being, including our values, abilities, experiences, successes, mistakes, justifications, and aspirations for both our personal and communal future. Through skilled storytelling, the seemingly chaotic complexities of our lives can be organized and contextualized, allowing us to better understand our place within both our personal microcosm and the larger societal narrative of which we are part (Bietti et al., 2019).

According to this logic, one of the most important advances in the study of personality from Freud and Adler to Allport and McAdams is the understanding that what makes us who we are depends not only on what we are, but also on what we tell ourselves we are (Karlsson et al., 2004). The process of personal development and growth is intrinsically linked to the ongoing exchange between our understanding of reality and the ways in which we represent it. Through such exchange, our understanding of reality is constantly constructed and reconstructed, simply by virtue of the act of describing it. This process of sense-making through narrative, both in our internal dialogue and in our communication with others, addresses a fundamental human need: the desire to attain a deep understanding of one’s own subjectivity and its role in the world (Cunliffe and Coupland, 2012).

And, in order to satisfactorily address this essential human need, the process of recounting our personal narrative must fulfill a specific set of requirements that collectively provide structure and coherence to the story. Baumeister has studied these issues for a long time, identifying, among other things, the four fundamental needs that the story of an existence must satisfy to produce a meaningful and complete vision (Baumeister, 1991; Baumeister and Newman, 1994; Baumeister and Vohs, 2002).

In any autobiography, whether implied or explicit, the primary component is a “purpose.” The significance of the events we encounter and the actions we undertake can only be comprehended in time and space when we can attribute a purpose to them that is capable of unifying what we have accomplished, who we have been, and what we are presently living. Such purpose may arise from the ideals of life, youthful aspirations, plans, and passions, as well as from painful turning points that may either open up new avenues or impede our path. The “purpose” generates significance by integrating the events and the decisions we make into an intelligible chain of causality, in a series of causes and effects through which we may attempt to account for our experiences.

But purpose is not enough, we also need “justification.” It is necessary that our personal story can “justify” what it describes. It is about the possibility of finding a rationale in what happens to us and what we do within a clear structure of values, a scheme that allows us to qualify events and actions as “right” or “wrong.” Whereas “purpose” generates meaning by framing events into a chain of causes and effects, justification does so by placing the facts of existence within a personal or collective moral code.

The third element of the plot is “effectiveness.” The possibility of reading our actions as capable of “making a difference,” of having an impact on what we consider good and of modifying the probability that what we wish comes true. Autonomy and a sense of control are integral parts of this meaning-generating function. Nothing can remove meaning and motivation from our actions more than the perception or awareness of the impossibility of changing things.

The last fundamental need that the narrative must be able to satisfy to generate meaning refers to the idea of “self-worth.” In the story of our existence, it is necessary for us to find reasons to describe ourselves as worthy of value and appreciation. Adam Smith had already grasped the importance of this aspect in his *Theory of Moral Sentiments* (1759/1976): “Man naturally desires, not only to be loved, but to be lovely; or to be that thing which is the natural and proper object of love. He naturally dreads, not only to be hated, but to be hateful; or to be that thing which is the natural and proper object of hatred. He desires, not only praise, but praiseworthiness; or to be that thing which, though it should be praised by nobody, is, however, the natural and proper object of praise. He dreads, not only blame, but blame-worthiness; or to be that thing which, though it should be blamed by nobody, is, however, the natural and proper object of blame. The love of praise-worthiness is by no means derived altogether from the love of praise. Those two principles, though they resemble one another, though they are connected, and often blended with one another, are yet, in many respects, distinct and independent of one another” (TMS III.2.1). Self-worth is, first and foremost, value *vis-à-vis* our own conscience.

The development of a personal narrative that satisfies these four fundamental human needs necessitates a continuous and unending process of assigning significance to one’s existence, including painful or troubling experiences, as well as those that fall at the margins of the “big story.” Crafting meaning through a narrative “plot” is especially critical for how individuals live their lives and cope with negative events and objective obstacles in their way. The ability to derive meaning from even the most traumatic and difficult experiences has a positive impact not only on psychological well-being, but also, somewhat unexpectedly, on physical health. In a study of subjects who suffered from heart attacks, Affleck et al. (1987) analyzed the patients’ reactions 7 weeks after the event, and then followed their course for the following 8 years. What emerges is that the majority of those who have been able to draw a positive lesson, a teaching, that is, those who have managed to find meaning in their painful and traumatic experience, 8 years after the event reported a better health condition, with a significantly lower probability of recurrence. Somewhat analogous results emerge from a study led by Bower et al. (1998) on subjects affected by HIV. After the communication of the diagnosis, individual reactions were different, but those who managed to experience the event with a constructive and positive attitude after the initial shock, that is, those who were able to attach a meaning to their story, report after many years a significantly lower mortality rate than those who failed to accept the experience of the disease. The ability to weave a meaningful plot about life adversities then represents a survival skill that helps to cope with the disappointments, conflicts, and suffering of human existence (Guo et al., 2013).

But it is appropriate to note that such narrative process is not devoid of potential hazards. There exists a constant risk to be misled by factors such as self-deception, overconfidence, and groundless optimism, as well as, in the opposite direction, a persistent sense of insatiable dissatisfaction. A key feature of the process of narrative construction of meaning is the interaction between our personal stories and the “big story” within which our existential plot line is set: the frames within which we are embedded, the driving and often conflicting rhetoric upon which they build, the visions of the future and the promises of the present that they suggest and imply. The “big story” represents the background of our small individual stories, like a choral canvas compared to the roles of the characters in comedy. However, it is essential to acknowledge that the characters in such narratives are not mere passive executors of a role assigned to them, but rather active contributors to the story’s development. Thus, the stories that we collectively tell ourselves about various aspects of our society, such as politics, the economy, and the future, are vital in shaping the plots of our lives. Like in literary works, we can distinguish between good and bad narrations in this “great social history,” with some being profound and enlightening while others superficial and failing to grasp the complexities of the human psyche. Therefore, it is crucial to recognize the importance of compelling narratives and their potential impact on our lives.

## 4. Meaning and work

Many workers deeply care about the meaning of their job and other non-monetary job attributes (Cassar and Meier, 2018). They are also willing to give up income for it (Hu and Hirsh, 2017; Maestas et al., 2018). However, it appears that meaningful jobs are becoming an increasingly scarce resource as demand continues to grow (Cotofan et al., 2023). “Could there be anything more demoralizing than having to wake up in the morning five out of 7 days of one’s adult life to perform a task that one secretly believed did not need to be performed—that was simply a waste of time or resources, or that even made the world worse?.” This was asked by the anthropologist David Graeber a few years ago in a very successful essay. And he continued: “Would this not be a terrible psychic wound running across our society? Yet if so, it was one that no one ever seemed to talk about” (2018, p. 8). The circumstances, especially after the COVID-19 epidemic, make a profound reflection on this point unavoidable today.

This “psychic wound running across our society”—as Graeber defines it—carries with it serious consequences. Jobs that are meaningless, useless, or even harmful, represent a huge waste not only of human resources, but also of natural and economic ones. A survey carried out by YouGov (2015) on a representative sample of British workers reported that 37% of them, with peaks of 41% in the London area, considered their work to be meaningless. Men (42%) were more likely to say their jobs were meaningless than women (32%). The survey also asked whether British workers found their jobs personally fulfilling, and 33% said they did not. Only 18% reported that their job was very fulfilling. Despite this, most people with meaningless jobs said it was unlikely that they would change jobs in the next 12 months (53%, compared to 35% who said they might change jobs). Multitudes trapped in meaningless jobs. In a 2020 follow-up survey (YouGov, 2020), a slightly better situation emerged, but still with concerning figures: 26% of respondents found their job “not very meaningful” or “not meaningful at all.” At the same time, 87% of them thought it was “very important” or “something important” that their work was meaningful. Another survey of 15,000 workers in ten European countries showed, among other things, that 22% of workers under 35 believed that their work was meaningless, 28% did not feel stimulated by what they did, and 27%, for the previous reasons, chose not to commit to it 100% (Deloitte, 2018). More recent data drawn from on a total of 122,416 respondents worldwide show that the percentage of employees “not engaged” or “actively disengaged” was in 2022 equal to 77%. Those “struggling” or “suffering” from daily negative emotions related to workplace are 65%; about one in three (29%) very often or always feels burned out at work, and, finally, one in two (51%) are actively looking for a different job (Gallup, 2023).

In another study Dur and van Lent (2019) find that, on a sample of 27,000 workers from 36 different countries, 17% of them report having serious doubts about their jobs having any social utility. When the data is disaggregated, we find that in the public sector the perception of the usefulness of one’s job seems to be generally higher than in the private sector (a significant difference of 6% on average). This is especially true, for example, for firefighters, police officers, social workers, the health professions, and teachers. For those who work in such sectors, the percentage of dissatisfaction with the meaning and purpose of one’s occupation is practically zero, with no significant difference between men and women, whereas those with higher educational qualifications tend to be slightly less satisfied, perhaps due to the greater risk of mismatch between field of study and specialization, and actual employment. A highly significant effect, however, is found as to age. Young people tend, on average, to consider their work comparatively less fulfilling in terms of meaning and social utility—an effect that could be due at least in part to the fact that younger generations are more deeply seeded in the post-scarcity frame than older ones. These data prompt us to think of a related question: what is the impact of a “wrong” job on our happiness? 77% of workers believe that having a socially useful job is important or very important and that, therefore, useless, or insignificant jobs negatively affect their subjective well-being. This is particularly true for those who believe that their job is socially useless: 96% of these agree that a job that allows them to be useful to others and to society is essential to feel satisfied with their life.

Regarding the relationship between job satisfaction and monetary compensation, it might be postulated that workers who perceive their job as meaningless could receive a higher wage as a sort of “uselessness premium” to compensate for their lack of job satisfaction. Dur and van Lent’s data show that there is no significant wage differential between satisfied and dissatisfied workers. Thus, workers who perceive their job as meaningless not only experience lower levels of job satisfaction but do not receive higher wages to cope with such disadvantage, at least in monetary terms. In this respect, Hu and Hirsh (2017), using a composite measure of “meaning,” which refers to the intrinsic interest in the job and the possibility of being useful to others, find that workers with a greater perception of job meaningfulness are less likely to accept alternative job offers, even if these offer higher wages. Meaning and purpose, therefore, are conducive to better worker satisfaction and attachment to work. If the job satisfies one’s need for meaning, people are willing to accept a relatively lower salary as compared to less meaningful alternative occupations. Conversely, if such need for meaning remains unmet, a higher salary will not compensate for the dissatisfaction.

In the light of the negative effects on personal well-being caused by “bullshit jobs,” as David Graeber puts it, why do we persist in creating them? Furthermore, why do we justify and legitimize an economic system that can render the lives of workers so meaningless? There are several hypotheses to consider. The first is a fact rather than a hypothesis: there are industries and jobs that do not create “goods,” but instead produce “bads.” Companies in these industries generate wealth for a few individuals while destroying value for many others. The so-called “sin industries” are among these, including tobacco, gambling, arms, and highly polluting companies. Additionally, companies with excessive market power or significant lobbying activities are often culpable. There is moreover a whole sector that Nobel laureates Akerlof and Shiller (2015) define as the “the economics of manipulation and deception” which systematically exploits consumers’ vulnerability, fragility, psychological weakness, and chronic lack of information, to make large profits at their expense. Being employed in such sectors is certainly not good for the workers’ need for meaning. The data provided by Dur and van Lent (2019) seems to point in this direction. Among the 20 worst jobs, when evaluated in terms of perceived social utility, there are, for example, sales, marketing, and public relations positions in finance and banking.

A second aspect that explains the existence of jobs that are perceived as meaningless, refers to the classic Marxian theory of alienation. It is the isolated, fragmented, and parceled nature of the job one is called to carry out, that makes work alienating and, therefore, unsatisfactory (Mottaz, 1981). There appears to be evidence supporting this explanation as well: among the least satisfying jobs Dur and van Lent (2019) indicate the assembler, the assembly line operator, the machine operator and all those positions that involve simple and repetitive manual activities.

Finally, a third source of “existential expropriation” is related to the way organizations are often designed, managed, and directed. We have, now, extensive evidence that shows how too vertical hierarchies elicit anomalous reactions on employees such as “psychic numbness” (Twenge et al., 2003) and that the prevailing ethos of management is control and not the more sustainable and desirable transfer of autonomy (Pink, 2011).

## 5. What makes a job a “good job”?

Having considered the main characteristics of a “bullshit job,” it is now appropriate to investigate the factors that confer greater value to an occupation and subsequently affect the level of satisfaction and well-being experienced by individual workers. Moreover, it is crucial to examine how the quality of work has transformed in recent times. Addressing these issues is essential to understand how the work sphere can contribute to the fundamental process of generating meaning and promoting human flourishing (Kim and Beehr, 2020).

Meaningful work is such when we can attach to it value, meaning and emotional involvement (Rothausen and Henderson, 2019). A meaningful job cannot be considered a luxury good for the few, but, rather, should be regarded as a basic human need (Yeoman, 2014). The impossibility of finding or attributing meaning to one’s working life can have far-reaching consequences on the well-being of individuals, organizations, and communities, more generally.

As to the point of what constitutes a “good” job, there are two distinct perspectives: one grounded in economics and the other in institutionalist and sociological frameworks. In the economic perspective, the quality of a job is ultimately determined by its wage. Assuming that every job involves disutility, the worker’s satisfaction reflects the extent to which the wage compensates for such disutility. A job may have various positive and negative attributes, and the worker will trade the negatives for a high wage and the positives for a lower wage – note however that the assumption that jobs typically have a net disutility implies that no job is intrinsically meaningful. Labor is then viewed as a commodity which, like all other commodities, comes at a price. The positive characteristics of a job will not increase its price, while the negative characteristics will. The difference between the wage and the net effect of the positive and negative attributes reflects, in this view, the job’s quality. This perspective may seem overly simplistic, and alternative approaches have been proposed by various parties, according to which the quality of a job depends on a mix of internal and external factors, such as the company’s culture, career prospects, job stability, training, the potential for professional growth, the role of the trade union, prevailing social and public policies, and the social recognition attached to the job, among others. Thus, the perception of any changes in the quality of the work are determined by a combination of economic, social, and political factors. In recent years, the job market has undergone many changes, and not all of them have led to improved job quality. A main trend characterizing the world of work in advanced economies in recent years seems to be an increased differentiation in job characteristics, which makes work more varied and diverse (Pistrui, 2018). For example, a worker in the automotive sector may now have, due to the reorganization and automation of assembly lines, a more creative and autonomous job, but salary and stability may be less satisfactory. On the other hand, teachers, who were once held in high esteem and largely autonomous in the choice of their educational approach, now experience increased administrative and bureaucratic demands and monitoring, a loss of trust from student families, and a reduction in social prestige.

All things considered, it appears that the quality of work has experienced a negative trend in recent decades (Howell and Kalleberg, 2019). Despite an improvement in wage levels and working hours, job stability and satisfaction seem to have significantly declined. It is generally observed that although average wages have risen and average working hours have decreased, the level of job satisfaction has either remained stable or decreased significantly depending on the countries considered. Data indicates that these changes are not traceable to individual values or characteristics but are rather linked to an increase in the stressful nature and instability of work. This trend has not been universal, as some workers, particularly those who are more highly educated, have been shielded from the worst effects of the decline in job quality, despite that they may nevertheless experience a decline in job satisfaction (Verhofstadt et al., 2007).

It is essential to recognize that the matter of job quality should not only be of concern to employees but also to employers, given the robust correlation between job satisfaction and productivity (Arnold et al., 2016). The provision of meaningful work is a necessary condition for the well-being of individuals, and thus, the establishment of conditions that ensure the presence of quality and meaning in the workplace holds significant ethical and moral implications (Koonmee et al., 2010).

A recent survey of over 500,000 workers in the United States and England was conducted by Bryce (2018) to analyze the primary non-monetary factors that determine workers’ well-being. The concept of “eudemonic” well-being, derived from Aristotle’s definition of eudaimonia, involves flourishing as a person and finding meaning and fulfillment in one’s life experiences. This raises the question of what factors contribute to the intrinsic value of a job. By analyzing data on time usage and associated well-being of British and American citizens, Bryce finds that jobs that satisfy our need for meaning and purpose typically have three common elements: a high level of professional autonomy, the ability to directly impact the well-being of others, and the opportunity to work within an organizational environment characterized by trust relationships. Thus, autonomy, pro-social impact, and trust are the ideal triad of elements for occupations that significantly impact our well-being. These elements can be thus be thought as the proper preventive cure for the “terrible psychic wound” caused by Graeber’s “bullshit jobs.” Cassar and Meier (2018), who surveyed and summarized a large body of research, used the expressions “workers’ autonomy in decision-making,” “feeling of competence,” and “worker’s feeling of relatedness” to refer to the same three elements, as did Martela and Riekki (2018). In a study involving a representative sample of the populations of 30 different European countries, Nikolova and Cnossen (2020) found that autonomy, competence, and quality of relationships are considered 4.6 times more important in assessing the significance of a job than opportunities for career advancement, working hours, monetary remuneration, and other forms of benefits.

Based on the above considerations, it can be inferred that viewing work solely as a source of disutility, with economic incentives as the only possible compensation, represents a gross misunderstanding with serious practical consequences. Humans are evidently more complex than this. Bryce (2018) shows that individuals working in non-profit, mission-oriented organizations with a social purpose derive greater meaning and satisfaction from their work than those engaged in profit-maximizing sectors. With regards to the role played by the sense of autonomy, self-employed workers experience higher levels of satisfaction, on average, compared to employees. The most accomplished workers, in terms of meaning, are those employed in social and community services, education, and healthcare. These individuals are also the most prone to burnout, however. Additionally, workers with higher levels of professionalism report higher levels of satisfaction than those with lower skills and competencies, even when greater responsibilities, stress, and fatigue are associated with their professional tasks. This negative impact appears to be offset by the positive effect associated with the attainment of greater autonomy, frequently linked to higher levels of skills and competencies. Finally, monetary incentives have a positive impact, albeit mainly when they are perceived as a form of recognition for the work performed, as opposed to a compensation for an insignificant and, as a result, unsatisfying activity.

## 6. Implications for organizational life

### 6.1. Creativity

The concept of creativity has often been naively characterized as an almost magical combination of inspiration and serendipity. However, research on creativity has shown that it is a multi-layered, intentional process that requires, among other things, significant effort and self-efficacy (Mathisen, 2011).

Contrary to the notion that creativity is associated with idleness, creative processes are typically very demanding in terms of mental focus and energy (Roskes et al., 2012). The creative process is in fact a complex mix of mind-wandering and mindful concentration (Agnoli et al., 2018), so that creative activity has generally little to do with the blissful, careless states ideally related to the notion of individual happiness. In a state of happiness, effortful concentration would be seen as a nuisance and be avoided. In a state of meaningfulness and flourishing, where tackling challenges is essential to the sense of purpose, effort on the contrary becomes part of the default mode of experience (Campbell et al., 2022). This difference is clearly seen in the creative trajectory of many highly successful content creators, e.g., star musicians. In the early phase of their career, they are typically very original and highly productive, and make creatively risky choices. As they get more accomplished, their creativity slows down and their output becomes less and less innovative, generally offering less and less surprising variations on their signature style. In a new situation of financial security and almost unlimited access to pleasurable, effortless alternatives, embarking again in a creative endeavor becomes less attractive, and when happening, there is a tendency to play safe rather than to break new, uncertain ground with the implied psychological costs and additional creative effort. There are clearly major exceptions to this pattern, but examples abound.

The relationship between financial incentives and creativity has been studied by Charness and Grieco (2019), who find that competitive incentives are effective in the case of closed creative tasks (where tasks have pre-determined goals and constraints) but not in the case of open tasks (where no specific restrictions apply), whereas non-monetary (but still competitive) incentives such as peer-ranking are effective for both kinds of tasks. Gross (2020) finds moreover that whereas a certain level of competitive incentives may stimulate people to be more creative (by exploring original and untested variations of their earlier work), excessive levels of competition block any creative effort. However, studying how incentives influence creativity does not give us direct insights as to how this contributes to job meaningfulness. Helzer and Kim (2019) explicitly link creativity to wellbeing on the workplace, and in particular to creativity as a coping resource enabling flexible responses to organizational stress. This result does not establish a direct link with meaningfulness yet but highlights how creativity may act as a shield against one of the main sources of job dissatisfaction, namely excessive stress. However, to fully appreciate the relationship between creativity at work and meaningfulness, we need to go beyond the study of creative behaviors as an instrumental response to incentives or as coping mechanisms, to consider how creativity is directly associated to sense of purpose on the job. And in this respect, we need to consider how creativity relates to what is typically considered a dis-incentive and that nevertheless surprisingly becomes a powerful motivational drive in this context: effort.

Recent research is highlighting a deep and so far, not sufficiently acknowledged connection between effort, value perception, and meaning. Results that are obtained through expenditure of effort tend to be considered more valuable by individuals (Inzlicht et al., 2018). This means that effort is not only a liability in terms of welfare comparisons but may become an important element of value creation, as in the well-known example of the so-called “IKEA effect” (Norton et al., 2012). Likewise, outcomes that are achieved through effort are regarded as more meaningful by individuals. This point becomes particularly interesting for the understanding of the relationship between meaning and creativity. The fact that creativity is also an effortful activity that requires considerable focus and persistence makes it especially valuable for individuals, who tend to consider it more meaningful than outcomes (e.g., in the case of creative processes, creative ideas) which are the result of non-effortful activity (e.g., mere copying of someone else’s idea). Creativity as a source of meaning need not be the result of individual processes: collective processes of creative thinking may be equally if not more meaningful and may also contribute to the group’s cohesion (Tang et al., 2020).

In work environments, stimulating creativity may become a powerful way to attach meaning to organizational roles and tasks (Carè et al., 2021). This is particularly clear in organizational environments where individuals are enabled to engage in creative processes that allow them to see such roles and tasks from different angles or to discover under-recognized dimensions and implications of their own agency. Through the creative process, individuals are invited to consider such agency not as merely abiding by a set of organizational norms and prescriptions, but as an opportunity to re-negotiate and re-think those aspects that are felt as particularly meaningless and alienating.

### 6.2. Job design

The need to carry out productive and meaningful work, to find profound meaning in our work activities seems to be like the air we breathe. We realize its importance only when it is polluted or begins to run out. Thus, today, we observe, on the one hand, an increasing awareness of the importance of the meaning of work and, on the other, a growing mistrust and disillusionment about the meaning and social usefulness of many jobs. And, paradoxically, we experience a growing demand for meaningful jobs together with a supply that seems, on the contrary, to gradually decrease. Sometimes it is the very nature of the job that makes it meaningless, in other cases it is the organizational environment in which the job takes place, the prevailing corporate culture or even the way in which the tasks are designed, and the workers are “managed”—which makes it difficult to find meaning and a satisfactory purpose in what we do. On both fronts something can and must be done, but it is on the aspects of a cultural and organizational nature that it is easier to intervene quickly without the need to wait for deep-seeded structural changes.

And this is for the good of the workers, but also for that of the organizations, because a job with meaning certainly generates more commitment, involvement, and motivation than a job considered useless or even socially harmful. Citing internal data from a 2005 research, David Fairhurst, then director of personnel at McDonald’s UK, argued that if the company had been able to “provide meaning” to its employees, 55% of them would have felt more motivated, 42% would have shown themselves more faithful and loyal and 32% more proud. “If something has value for people then it has meaning—Fairhurst told People Management—and this can be created by giving workers a sense of a common purpose” (cited in Overell, 2008).

However, that management is often part of the problem rather than the solution is demonstrated, not without a certain irony, also by the fact that Fairhurst himself was fired a few years later because, as reported by some colleagues, he made women he worked with feel “not really comfortable” (Haddon, 2020). This episode also raises the question of what are the organizational factors that enable the creation of a culture, a climate and practices that facilitate the generation of meaning: as we said, mainly “autonomy,” “relationality” and “social purpose.” A further element that enriches this picture and which should increasingly characterize relationships within organizations is what we can define as “respect.” Organization psychologists Cleveland et al. (2015) wrote a paper titled “The future of HR is RH,” claiming that the future of human resource management (HR) lies in the respect that is owed to every human being (RH) also and perhaps mainly, in the workplace. The English expression “paying respect” normally used to as equivalent to “to respect,” “to consider,” “to have regard,” suggests, even etymologically, that respect is a precious currency; a factor that can, therefore, act as a powerful source of motivation. Receiving appreciation for the work done is, among all the factors that cause satisfaction, the only one that always appears among the first two most important items mentioned by workers in all the surveys carried out during 50 years into the post-war period. Other values change: monetary remuneration, job security, career opportunities, have acquired or lost relative importance over the years, but feeling respected for the fruit of one’s work has invariably remained among the most important elements in determining worker satisfaction (Wiley, 1997).

How can an organization launch processes that promote feelings of respect among its members while avoiding the perception of intrusiveness and instrumentalism? Recent years have seen economists gather compelling evidence on this topic.

#### 6.2.1. Acknowledgement and awards

The first aspect has to do with symbolic rewards. Rewards, unlike incentives, are public awards, without great intrinsic monetary value, and are not automatic. Incentives, on the other hand, are private, economically substantial and are assigned on a contingent basis. Numerous studies have shown that the use of monetary incentives can create a sense of control and reduced responsibility, leading to unintended consequences such as reduced performance instead of the desired improvement. Awards, on the other hand, due to their public visibility and their symbolic nature convey a message of approval and social recognition that enhances and strengthens motivation. Symbolic rewards have a more significant impact when they acknowledge not only the individual’s work but also the team’s efforts. In this case, the organization communicates that both the outcome and the collaborative process that led to it are valuable. Profit-sharing schemes often hold little monetary value for most workers. However, they can serve as effective symbolic rewards, perhaps precisely because of this reason (Frey, 2006; Neckermann and Frey, 2013; Gallus and Frey, 2016).

#### 6.2.2. The gift of attention

The second ingredient of respect is the “gift of attention.” The gift we make to others of our time, our cognitive resources, our memory, and our commitment. Being attentive to others means, first, being available and then being able to put oneself in the shoes of others, to mentalize, that is, to look at the world with their eyes and to empathize, that is, to let oneself be infected by their emotional states: to suffer together, rejoice together, using Adam Smith’s concept of “fellow-feelings.” The so-called “Hawthorne experiments” were originally meant as one of the first attempts to scientifically study the effect of the physical environment on labor productivity. The first variable considered was the level of illumination in a Western Electric Company factory near Chicago. After long observation, the researchers concluded that productivity increased both in the event of an increase in the brightness of the environment and in the case in which it was reduced. No definite interpretative model emerged, until the experimenters decided to interview the workers and it was understood, then, that the reason why labor productivity had increased was linked to the workers’ response to the attention they were receiving from the company. They had perceived the experiment as an attempt to improve their working conditions and, regardless of any effective environmental changes, they had been motivated to work harder and better, as a sign of gratitude for such attention. In particular, the participants in the experiments were given more freedom to determine the conditions of their working environment and to set their own production standards. In the interaction with the other workers involved, cooperation and group cohesion had intensified. Ultimately, it was understood that satisfaction and performance at work depended more on cooperation and the perception of self-worth than on physical working conditions. The issue highlighted by Hawthorne’s experiments and the interpretation given by the psychologist Elton Mayo, was that managers customarily thought that the answers to industrial problems lay mainly in technical efficiency, whereas the answer was related to human and social factors (Mayo, 1933); see also Levitt and List (2011). Even today, when we talk about the “Hawthorne effect,” we refer to the fact that the simple participation in an experiment can modify the behavior of the subjects, independently of any manipulation by the researchers. But the mechanism that hides under the “Hawthorne effect” is deeper and originates from our sensitivity to relationships and especially to those in which we receive attention.

#### 6.2.3. The importance of being trusted

The third ingredient of respect is trust. That vulnerable trust which, precisely because it places those who trust in a condition of risk and exposes them to opportunism and betrayal, contributes to generate a reliable response. It is the mechanism of “trust responsiveness” (Pelligra, 2005, 2010, 2011). Trust, even when there is a less risky alternative, increases trustworthiness. Being open to trust also pays off in the organization. This does not mean that there are no cases of opportunism and moral hazard. The point is whether in an organization based on trust, reliable behaviors outnumber opportunistic ones in frequency and meaningfulness. Distrust protects against betrayal, but at the same time generates closure and resentment. Trust exposes to betrayal but elicits trustworthiness and cooperation. The experimental and field evidence seem to converge in showing that the benefits of the latter type far outweigh the costs.

In a nutshell, we can conclude that symbolic rewards, attention to the relationship and responsive trust represent the key elements upon which a relationship of respect can be built.

## 7. Conclusion

Meaningfulness and sense of purpose are key elements in determining job satisfaction and in enabling dedicated, productive work. However, economic theory has for a long time adopted a very limited characterization of work as an activity carrying net dis-utility, that is, devoid of intrinsic value and meaning and only worthwhile if compensated by a monetary reward. There is substantial evidence showing instead that human work motivation is driven by perceptions of self-worth, agency, trust, and respect, among others. Despite that recent research has clearly expanded our understanding of such phenomena, much remains to be done in terms of better appreciation of the actual biobehavioral pathways through which such perceptions generate states of well-being and flow. Likewise, still very little is known about how such strong intrinsic motivation and the consequent dedication to job challenges and related effort provision may cause workers to be more exposed to undesirable outcomes such as excessive stress and even burnout. The possibility of imbalances between effort and reward, not only monetary but also symbolic, may in particular cause workers who are more susceptible to stress on the job to work harder while attaining lower subjective well-being (Cottini et al., 2023). Designing work environments and compensation schemes that keep such factors fully into account is a key, high-stakes challenge for future research and policy. Success or failure may have major consequences for workers well-being, economic productivity, and collective welfare.

## Author contributions

All authors listed have made a substantial, direct, and intellectual contribution to the work and approved it for publication.

## Funding

This work was supported by the University of Cagliari and Fondazione di Sardegna research project “B.ECO.ME.—Behavioral Economics of Meaning,” CUP: F75F21001500007.

## Conflict of interest

The authors declare that the research was conducted in the absence of any commercial or financial relationships that could be construed as a potential conflict of interest.

## Publisher’s note

All claims expressed in this article are solely those of the authors and do not necessarily represent those of their affiliated organizations, or those of the publisher, the editors and the reviewers. Any product that may be evaluated in this article, or claim that may be made by its manufacturer, is not guaranteed or endorsed by the publisher.
